# Supplementary material for: Machilin D, a Lignin Derived from Saururus chinensis, Suppresses Breast Cancer Stem Cells and Inhibits NF-κB Signaling
Source: Biomolecules. 2020 Feb 5;10(2):245. doi: 10.3390/biom10020245 (PMC7072518; doi:10.3390/biom10020245)
Supplement: Supplementary file 1 [file biomolecules-10-00245-s001.pdf]

Table S1. Specific Real-time RT-qPCR primer sequences containing *IL-6, IL-8, and β-actin* genes

Genes	Primers
**IL-6**	Forward: 5’-AGACAGCCACTCACCTCTTCAG-3’<br>Reverse: 5’-TTCTGCCAGTGCCTCTTTGCTG-3’
**IL-8**	Forward : 5′-ATGACTTCCAAGCTGGCCGT-3′,<br>Reverse : 5′-TCCTTGGCAAAACTGCACCT-3′
**β-actin**	Forward: 5’-TGTTACCAACTGGGACGACA-3’<br>Reverse : 5’-GGGGTGTTGAAGGTCTCAAA-3

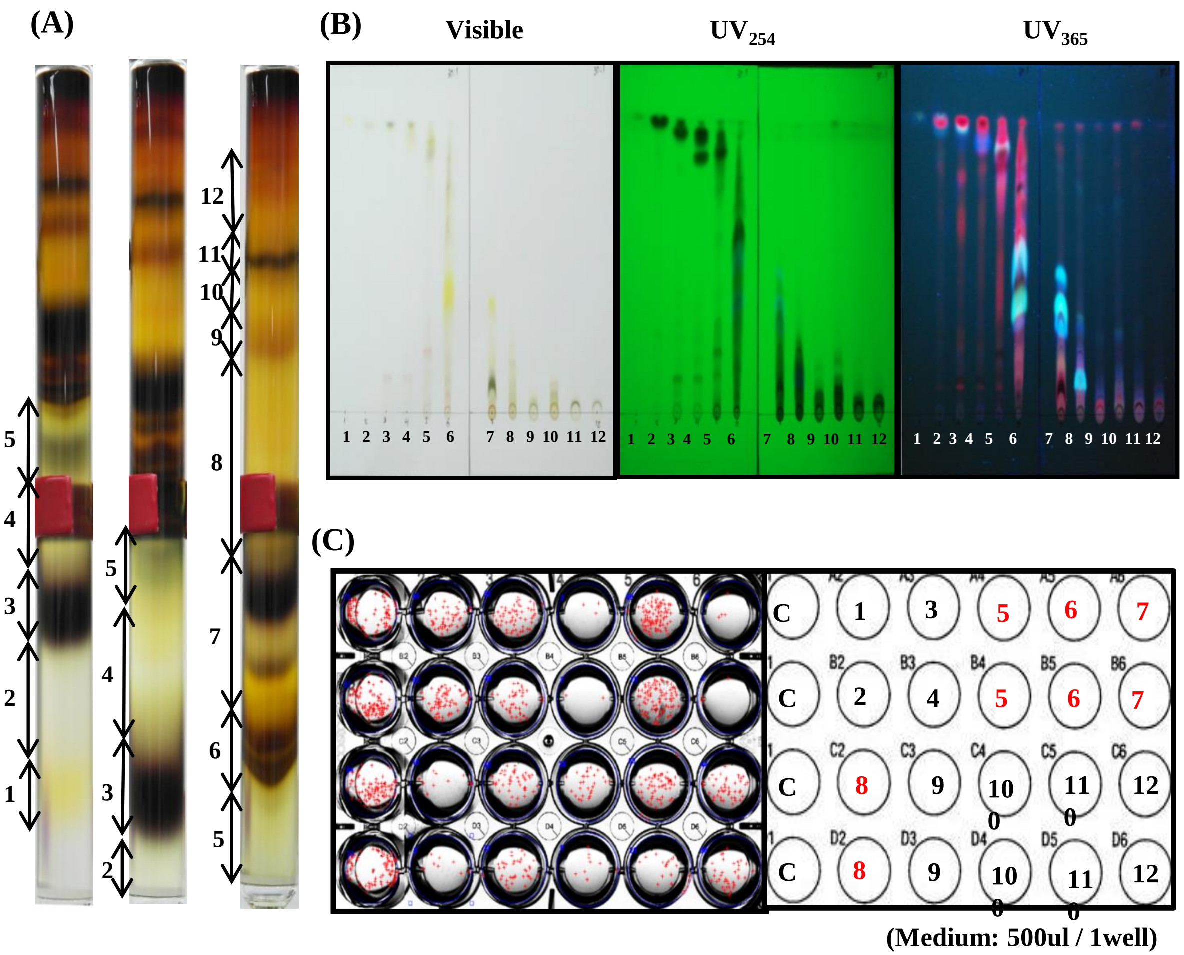

Supplementary Figure S1. The purification procedure of cancer stem cell inhibitor derived from *S. chinensis* using SiO_2 gel chromatography. (A) Sample was isolated by using SiO_2 gel chromatography with solvent mixture $CHCl_3$: MeOH = 30:1 (B) TLC plate's analysis of the purified sample. The eluated fractions were spotted on TLC plate and developed in solvent-saturated chamber in $CHCl_{3:}$ MeOH = 30:1. The TLC plates were dried, and the spots on TLC plates were analyzed and detected under UV lamp. (C) Mammospheres formation assay using breast cancer stem cell derived from MCF-7 cells. Cells were treated with 1μl and 2μl of each fraction. After a week incubation (5% CO2, humidified incubator at 37°C, 7 days), the mammosspheres were scanned and counting the tumorspheres in NICE program.

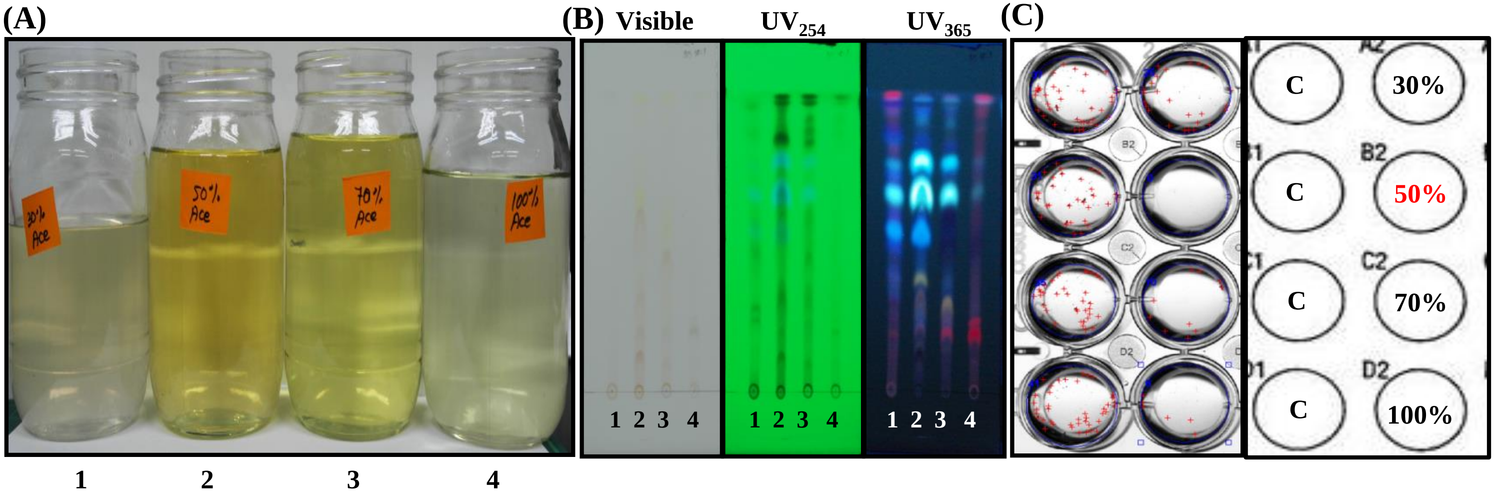

Supplementary Figure S2. Purification procedure of cancer stem cell inhibitor in *Saururus chinensis* using Reversed-Phase C_{18} (ODS) gel choromatography. (A) ODS open column chromatography is eluted with 30%, 50%, 70% and 100% of CH_3CN using partial-purified sample. (B) Thin-layer chromatography analysis of the partial-purified sample from ODS resin, purified-sample were spotted and developed in chloroform: methanol *(*30:1). UV was used to detect the samples. (C) Mammosphere formation assay using breast cancer stem cell derived from MCF-7 cells using partial-purified.

(A)

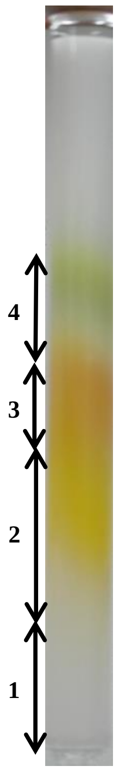

(B)

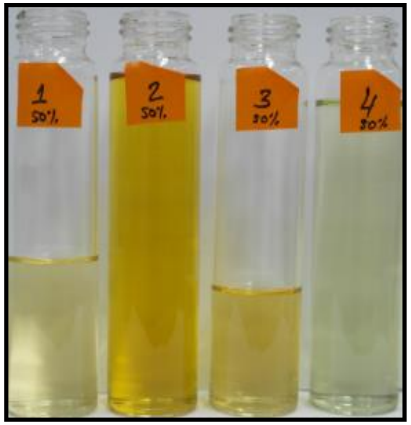

Fra. No	50% Acetonitrile		MeOH Stock
	Solvent	Quantity	
1.	MeOH	27ml	1.5ml
2.	MeOH	65ml	3ml
3.	MeOH	21ml	1.5ml
4.	MeOH	109ml	1.5ml

(C)

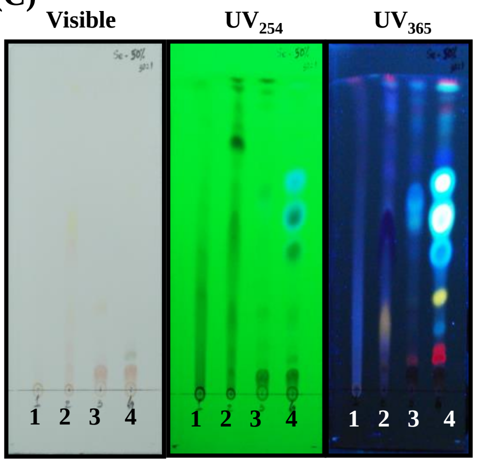

(D)

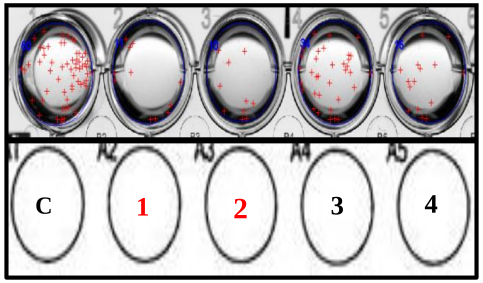

(Medium: 500ul / 1well)

Supplementary Figure S3. Purification procedure of cancer stem cell inhibitor in *Saururus chinensis* using Sephadex LH-20 gel chromatography. (A) Sephadex LH-20 column chromatography eluted with methanol. (B) Image of sample's color and table of the sample fractions. (C) Thin-layer chromatography analysis of the eluted fractions of *Saururus chinensis* which developed in chloroform/methanol (30:1). (D) Mammosphere formation assay using breast cancer stem cell derived from MCF-7 cells using partial-purified sample.

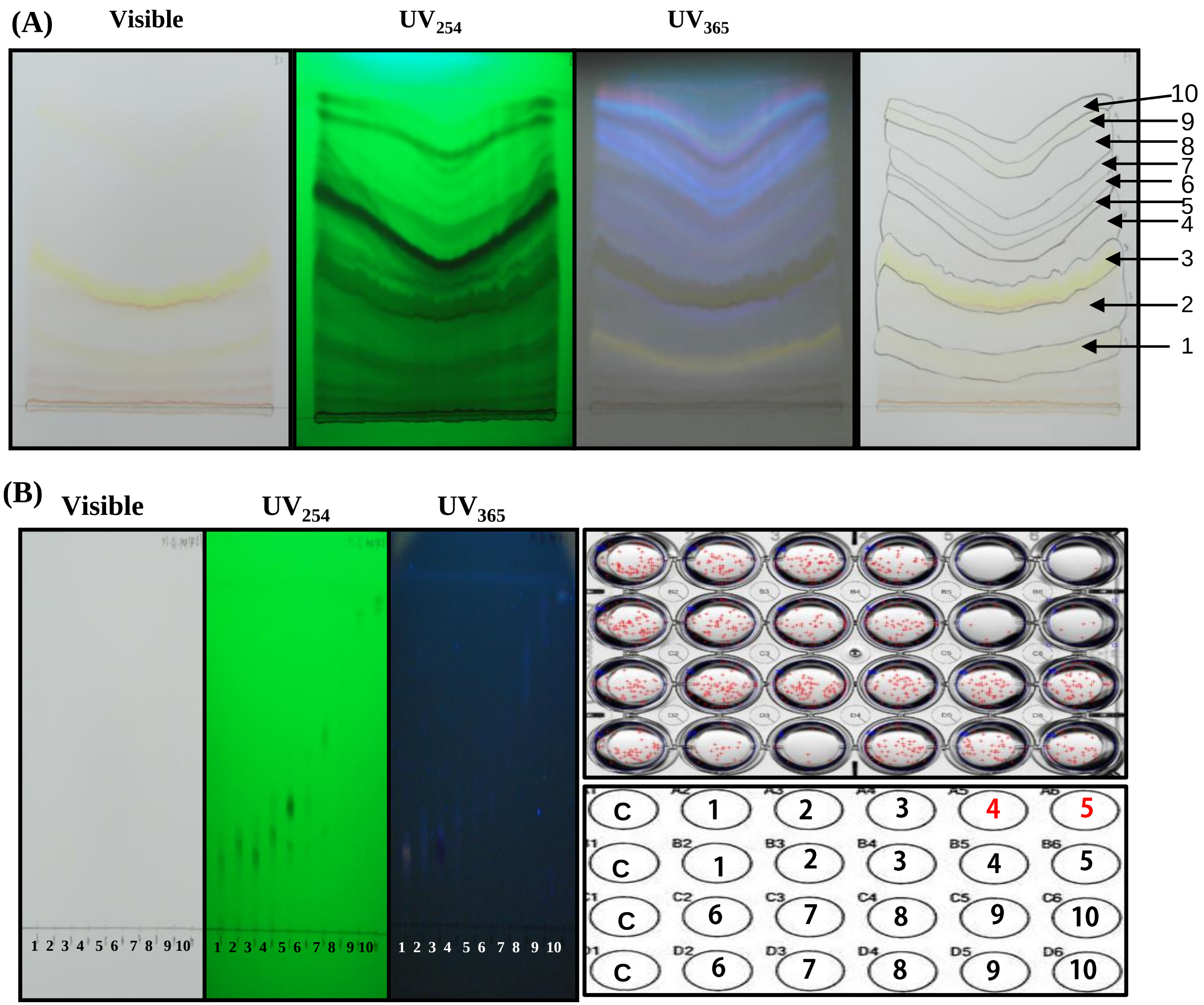

Supplementary Figure S4. Purification procedure of cancer stem cell inhibitor in *Saururus chinensis* using a preparative thin layer chromatography (TLC). (A) Preparative Thin Layer Chromatography of partial-purified sample. (B) Thin layer chromatography analysis of the Prep-TLC fractions which developed in chloroform/methanol *(*30:1). (C) Mammosphere formation assay using breast cancer stem cell derived from MCF-7 cells using partial-purified sample.

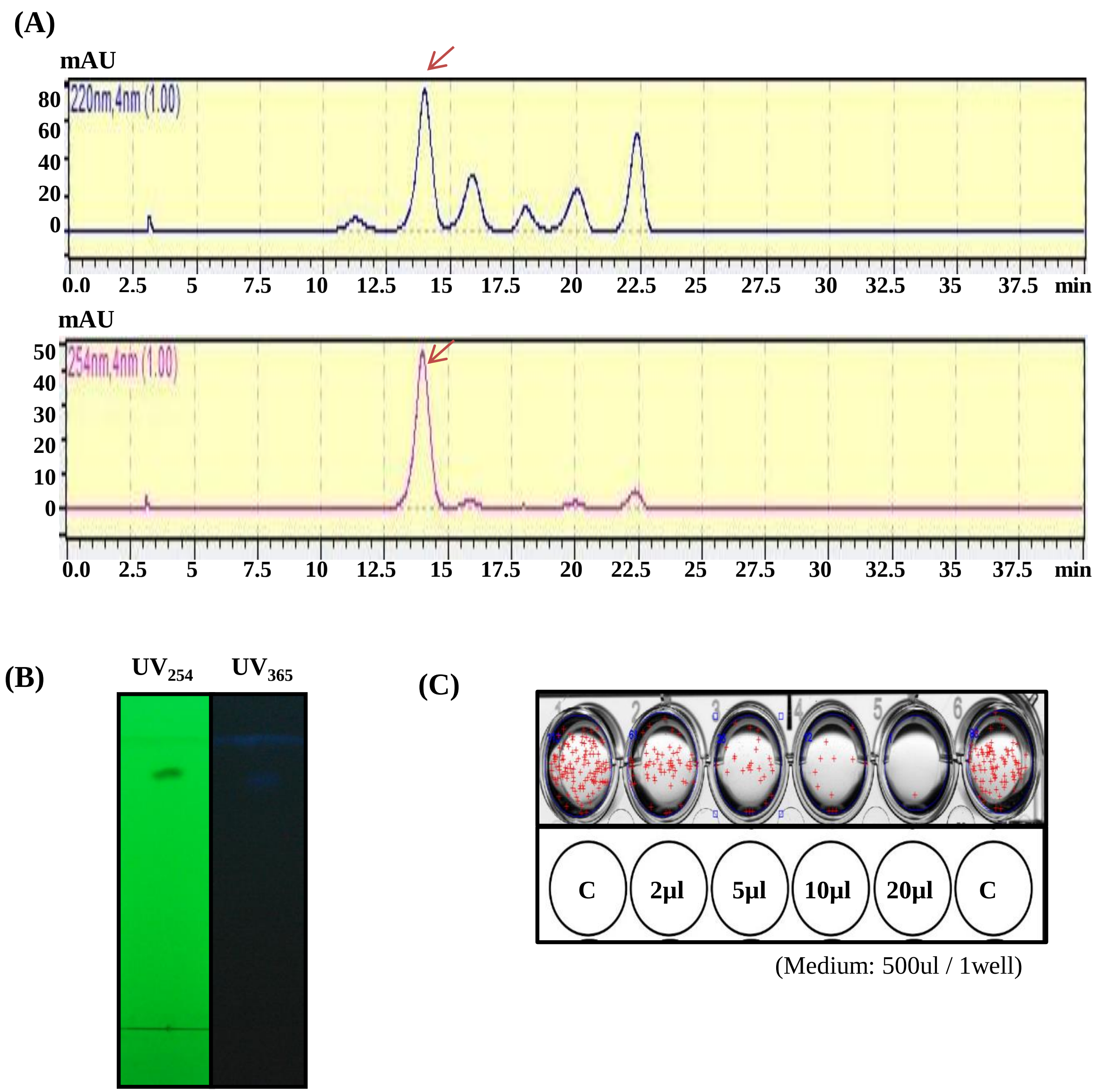

Supplementary Figure S5. Major fraction collected by using High Performance Liquid Chromatography in two wavelength. Sample was collected based on 254 nm wavelength. (A) Upper figure; HPLC chromatogram was detected in UV$_{220nm}$. Down figure; HPLC chromatogram was detected in UV$_{254nm}$. (B) Thin-layer chromatography analysis of the HPLC purified sample. (C) Mammosphere formation assay using breast cancer stem cell derived from MCF-7 cells using partial purified sample.

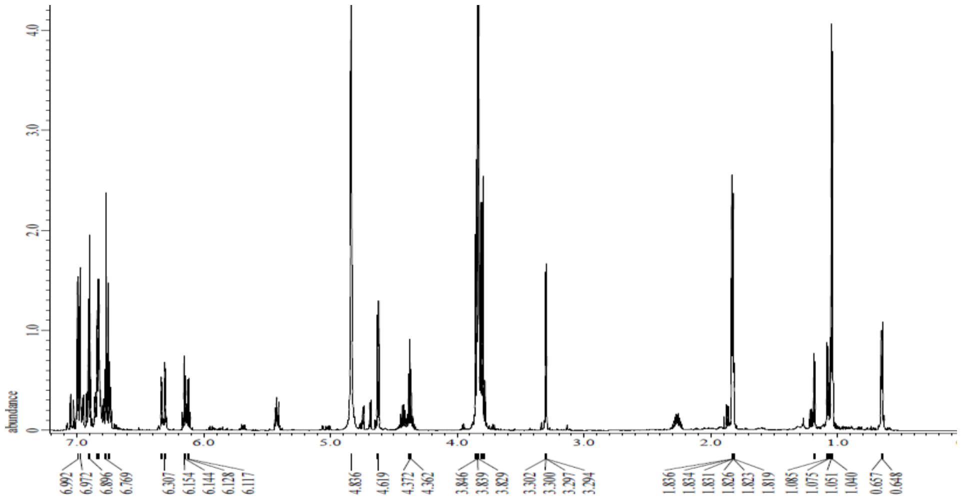

^{1}H-NMR spectrum

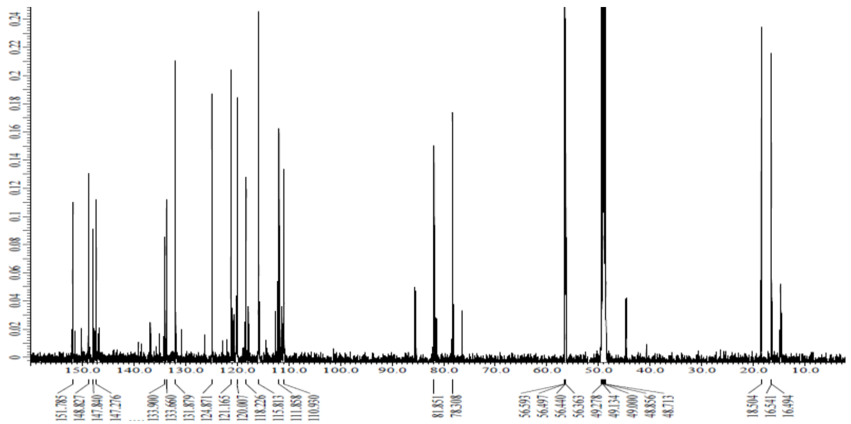

^{13}C-NMR spectrum

Supplementary Figure S6. ^{1}H NMR and ^{13}C NMR spectra of the purified sample

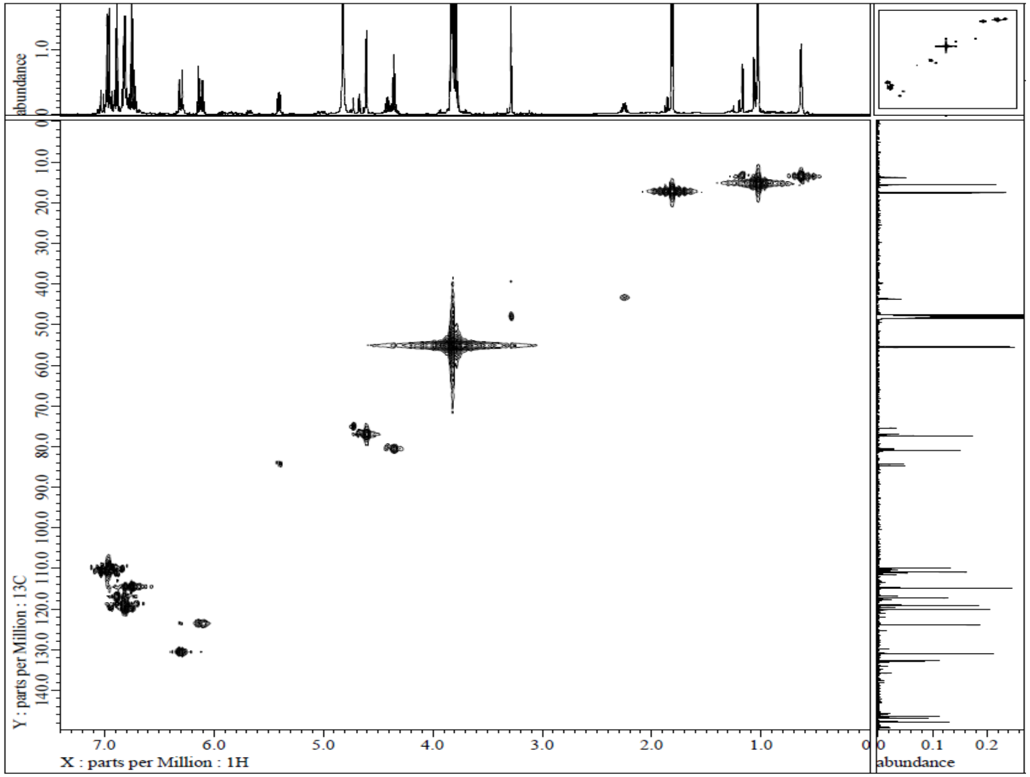

Supplementary Figure S7. HMQC spectrum of the purified sample

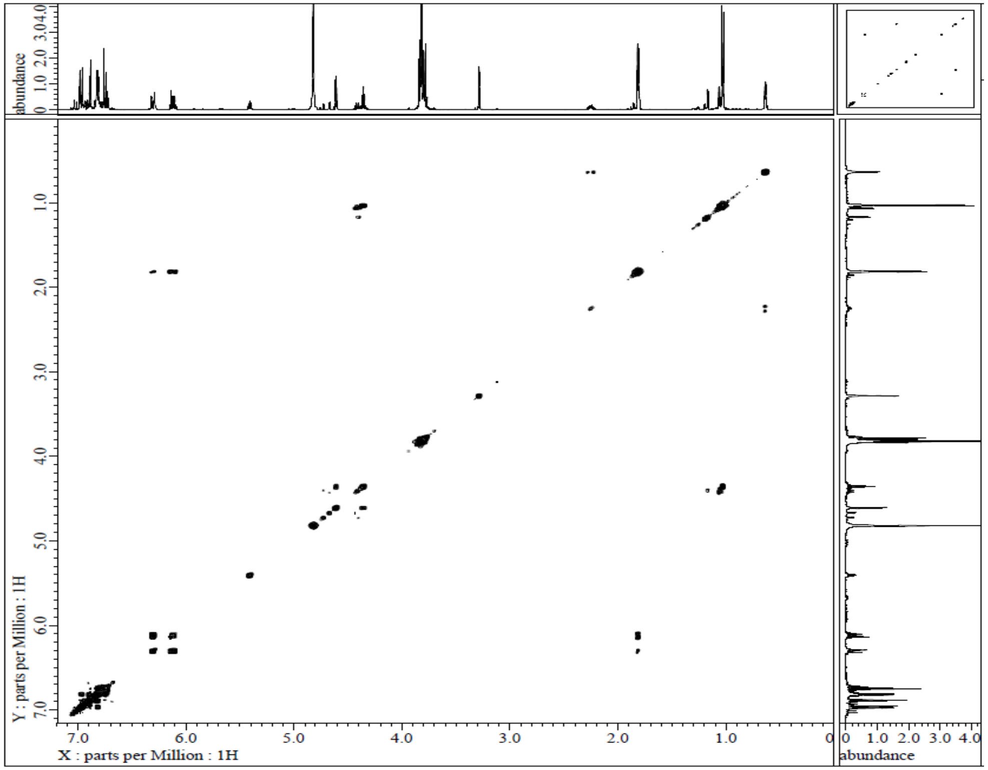

Supplementary Figure S8. ^{1}H-^{1}H COSY spectrum of the purified sample

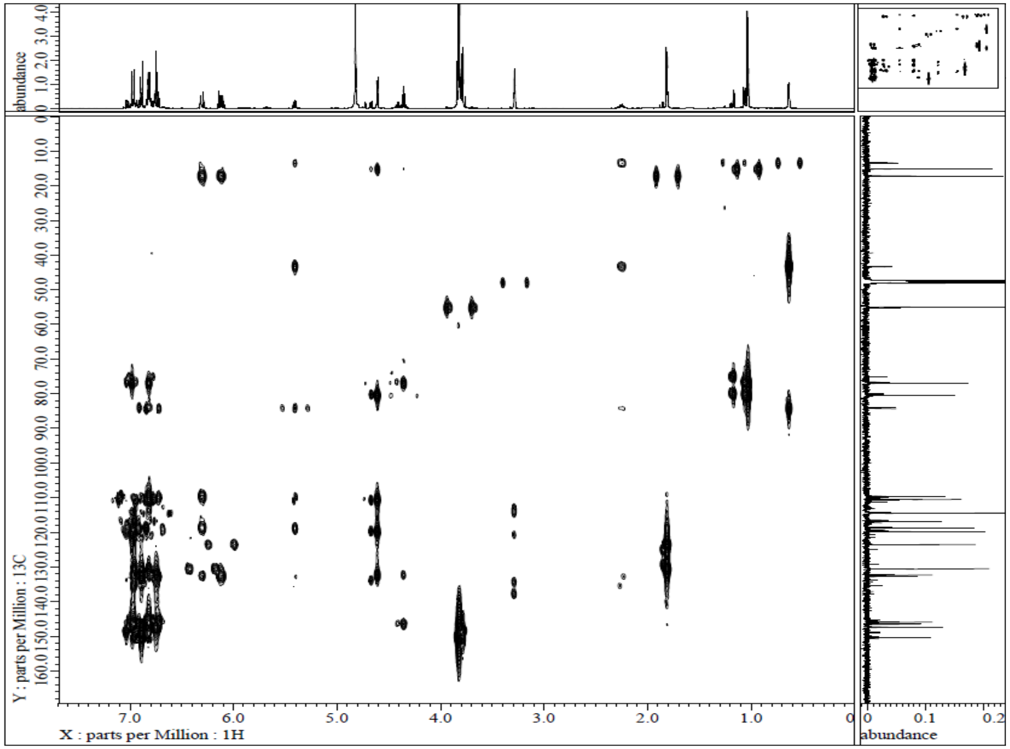

Supplementary Figure S9. HMBC spectrum of the purified sample

(A)

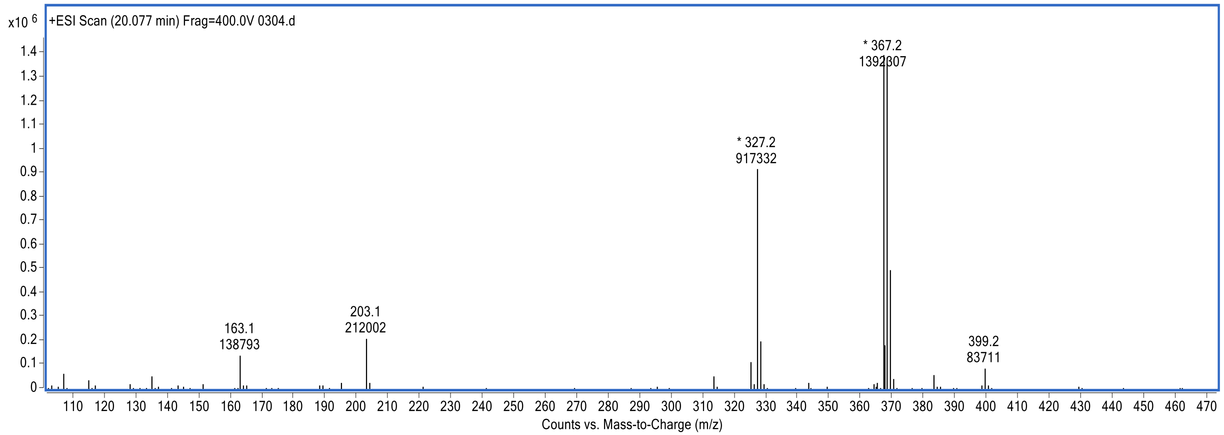

(B)

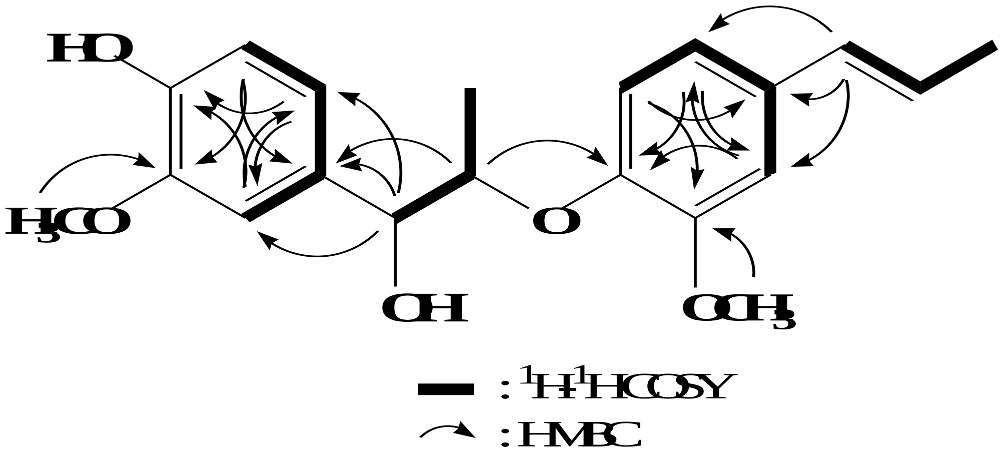

Supplementary Figure S10. ESI mass spectrometry (A) and two-dimensional NMR data (B) of the purified sample
